# Brazilian Thoracic Association recommendations for the management of lymphangioleiomyomatosis

**DOI:** 10.36416/1806-3756/e20240378

**Published:** 2025-01-31

**Authors:** Bruno Guedes Baldi, Paulo Henrique Ramos Feitosa, Adalberto Sperb Rubin, Alexandre Franco Amaral, Carolina Salim Gonçalves Freitas, Cláudia Henrique da Costa, Eliane Viana Mancuzo, Ellen Caroline Toledo do Nascimento, Mariana Sponholz Araujo, Marcelo Jorge Jacó Rocha, Martina Rodrigues de Oliveira, Tatiana Senna Galvão, Pedro Paulo Teixeira e Silva Torres, Carlos Roberto Ribeiro Carvalho

**Affiliations:** 1. Divisao de Pneumologia, Instituto do Coracao - InCor - Hospital das Clinicas, Faculdade de Medicina, Universidade de São Paulo - HCFMUSP - São Paulo (SP) Brasil.; 2. Hospital da Asa Norte, Brasília (DF) Brasil.; 3. Serviço de Pneumologia, Pavilhão Pereira Filho, Santa Casa de Porto Alegre, Porto Alegre (RS) Brasil.; 4. Hospital AC Camargo, São Paulo (SP) Brasil.; 5. Universidade do Estado do Rio de Janeiro, Rio de Janeiro (RJ) Brasil.; 6. Serviço de Pneumologia e Cirurgia Torácica, Hospital das Clínicas, Universidade Federal de Minas Gerais, Belo Horizonte (MG) Brasil.; 7. Departamento de Patologia, Hospital das Clinicas, Faculdade de Medicina, Universidade de São Paulo - HCFMUSP - São Paulo (SP) Brasil.; 8. Divisão de Pneumologia, Hospital de Clínicas, Universidade Federal do Paraná, Curitiba (PR) Brasil.; 9. Hospital de Messejana, Fortaleza (CE) Brasil.; 10. Hospital Universitário Professor Edgar Santos, Universidade Federal da Bahia, Salvador (BA) Brasil.; 11. Hospital Israelita Albert Einstein, Goiânia (GO) Brasil.

**Keywords:** Lymphangioleiomyomatosis/diagnosis, Lymphangioleiomyomatosis/prevention & control, Lymphangioleiomyomatosis/pathophysiology, Lymphangioleiomyomatosis/drug treatment, Lymphangioleiomyomatosis/therapy, Clinical practice guide, Linfangioleiomiomatose/diagnóstico, Linfangioleiomiomatose/prevenção & controle, Linfangioleiomiomatose/fisiopatologia, Linfangioleiomiomatose/tratamento farmacológico, Linfangioleiomiomatose/terapia, Guia de prática clínica

## Abstract

Lymphangioleiomyomatosis (LAM) is a rare disease, characterized as a low-grade neoplasm with metastatic potential that mainly affects women of reproductive age, in which there is proliferation of atypical smooth muscle cells (LAM cells) and formation of diffuse pulmonary cysts. It can occur in a sporadic form or in combination with tuberous sclerosis complex. In recent decades, a number of advances have been made in the understanding of the pathophysiology and management of LAM, leading to improvements in its prognosis: identification of the main genetic aspects and the role of the mechanistic target of rapamycin (mTOR) pathway; relationship with hormonal factors, mainly estrogen; characterization of pulmonary and extrapulmonary manifestations in imaging studies; identification and importance in the diagnosis of VEGF-D; a systematic diagnostic approach, often without the need for lung biopsy; use of and indications for the use of mTOR inhibitors, mainly sirolimus, for pulmonary and extrapulmonary manifestations; pulmonary rehabilitation and the management of complications such as pneumothorax and chylothorax; and the role of and indications for lung transplantation. To date, no Brazilian recommendations for a comprehensive approach to the disease have been published. This document is the result of a non-systematic review of the literature, carried out by 12 pulmonologists, a radiologist, and a pathologist, which aims to provide an update of the most important topics related to LAM, mainly to its diagnosis, treatment, and follow-up, including practical and multidisciplinary aspects of its management.

## RESUMO

A linfangioleiomiomatose (LAM) é uma doença rara, caracterizada como uma neoplasia de baixo grau, com potencial metastatizante, que atinge principalmente mulheres em idade reprodutiva, em que se evidencia proliferação de células musculares lisas atípicas (células LAM) e formação de cistos pulmonares difusos. A LAM pode ocorrer na forma esporádica ou associada ao complexo de esclerose tuberosa. Vários progressos ocorreram no entendimento da fisiopatologia e no manejo da LAM nas últimas décadas, determinando melhora do seu prognóstico, incluindo: identificação dos principais aspectos genéticos e do papel da via da proteína alvo mecanístico da rapamicina (mTOR); relação com fatores hormonais, principalmente estrogênio; caracterização das manifestações pulmonares e extrapulmonares em exames de imagem; identificação e importância no diagnóstico do VEGF-D; abordagem diagnóstica sistematizada, muitas vezes sem necessidade de biópsia pulmonar; uso e indicações dos inibidores de mTOR, principalmente sirolimo, para quadros pulmonares e extrapulmonares; reabilitação pulmonar, abordagem de complicações, como pneumotórax e quilotórax; e papel e indicações do transplante pulmonar. Não havia até o momento uma publicação nacional com recomendações para a ampla abordagem da doença. Este documento se caracteriza como uma revisão não sistemática da literatura, realizada por 12 pneumologistas, um radiologista e um patologista, que visa atualizar os tópicos mais importantes relacionados principalmente ao diagnóstico, tratamento e seguimento da LAM, incluindo aspectos práticos e multidisciplinares do seu manejo.

## INTRODUCTION

Lymphangioleiomyomatosis (LAM) is a rare disease classified as a low-grade, multisystemic, progressive metastatic neoplasm, characterized by the proliferation of atypical smooth muscle cells (LAM cells) around blood and lymphatic vessels, as well as airways, manifesting as the formation of diffuse pulmonary cysts.^1^ ^,^ ^2^ The site of origin of LAM cells remains unknown. Individuals with LAM can develop tumors, such as angiomyolipomas and lymphangioleiomyomas.^2^ ^,^ ^3^

Caused by mutations in the tuberous sclerosis complex (TSC) genes *TSC1* and *TSC2*, LAM mainly affects women of reproductive age. It occurs in sporadic forms or in association with TSC. It is a genetic disease characterized by multiple benign tumors of the skin, central nervous system, retina, heart, liver, kidneys, and lungs.^3^ ^-^ ^5^

In recent years, there have been several advances in the understanding of and approach to LAM, including its pathophysiology, functional behavior, response to exercise, diagnosis, and treatment, resulting in improved prognosis and management.

The importance of preparing this document is underscored by the following factors: the increase in the number of diagnosed cases of LAM, especially with the expansion of access to chest CT; the need to expand knowledge about the disease among pulmonologists, clinicians, and specialists in other areas, in order to reduce its underdiagnosis; the absence of Brazilian recommendations for a comprehensive approach to the disease; the presence of a very active patient association (the Brazilian Association of Individuals with Lymphangioleiomyomatosis), which has helped obtain numerous benefits for individuals with the disease, including access to centers of excellence and to treatment; and the need to emphasize the importance of a preferably multidisciplinary approach to LAM, given its multisystemic nature.

Twelve pulmonologists, one radiologist, and one pathologist with extensive experience in the subject were brought together to prepare this document. A non-systematic narrative review of the literature was carried out, and the main existing evidence on various topics was included, including practical aspects of the management of the disease.

## EPIDEMIOLOGY

The fact that LAM is a rare disease that is poorly understood and underdiagnosed often delays its treatment. Studies of LAM have largely been retrospective and many have included cases of LAM accompanied by TSC (LAM-TSC).^6^ However, in recent years, an increase in the prevalence of LAM has been observed in several studies, possibly reflecting advances in the ability to recognize the disease, including increased access to chest CT. Approximately 25 years ago, the prevalence of LAM in various locations, including the United Kingdom, France, and the United States, was 1 case per million women.^7^ ^-^ ^9^ A subsequent study that evaluated the prevalence of LAM in countries on different continents identified a prevalence of 3.4-7.8 cases per million women.^6^ In a more recent study, the prevalence of LAM in four European countries was estimated at 23.5 cases per million adult women and 19.0 cases per million women of all ages,^10^ substantially higher than previous estimates. Relevant factors in that later study include assessments at well-structured national centers in countries with smaller populations, optimizing the chances of identifying the disease.^10^ Estimates from the LAM Foundation indicate a prevalence of 3-5 cases per million women.^11^ Although there are no reliable data on the prevalence of LAM in Brazil, it is estimated, on the basis of the most recent studies conducted elsewhere,^6^ ^,^ ^10^ ^,^ ^11^ that there are 1,000-2,000 patients in the country.

Most patients presenting with LAM are premenopausal, in their third or fourth decade of life; however, the age range extends from preadolescence to old age.^3^ ^,^ ^12^ Only one study demonstrated ethnic variability, suggesting that sporadic LAM was more common in White women of higher socioeconomic status, although that finding might be attributable to biases in access to health care.^12^

The rates of LAM are highest in patients with TSC, a condition with an incidence of approximately 1 in 5,000-10,000 live births.^5^ The frequency of LAM in women with TSC is reported to be 26-50%, with higher rates among those over 15 years of age, ranging from 27% in those under 21 years of age to 80% in those over 40 years of age.^12^ ^-^ ^14^ Although LAM can occur in men with TSC, the sporadic form is extremely rare in such men.^14^ In men with TSC, the reported frequency of cystic lung disease ranges from 10% to 38%, although the development of symptoms and a decline in lung function are uncommon.^14^ ^,^ ^15^

## PATHOPHYSIOLOGY

The pathophysiology of LAM is complex, involving multiple mechanisms, and is still not fully understood, despite significant advances in recent years. Mutations in the tumor suppressor genes *TSC1* and, more commonly, *TSC2* are associated with the development of LAM. In LAM-TSC, those mutations are present in the germline and it is assumed that a second somatic mutation occurs in the tissue (second hit), leading to a loss of heterozygosity. In sporadic LAM, the mutations are present in somatic cells and are identified in various tissues, including the lungs, kidneys, and lymph nodes.^16^

The *TSC1* and *TSC2* genes encode, respectively, the proteins hamartin and tuberin, which form the hamartin-tuberin complex, responsible for inhibiting the mechanistic target of rapamycin (mTOR). The mTOR pathway is part of a complex protein synthesis pathway (P13K/mTOR/AKT) through the mTORC1 and mTORC2 protein complexes. Tuberin deactivates the Rheb protein, which in turn deactivates the mTORC1 pathway, which is responsible for several functions of protein synthesis, cellular metabolism, and angiogenesis. Through mutations in the *TSC1* and *TSC2* genes, this inhibitory effect on the mTOR pathway is lost, and that pathway becomes hyperactivated, resulting in the growth, proliferation, and dissemination of LAM cells.^17^

The etiology of LAM cells is unclear. The genetics, immunohistochemical profile, and morphological pattern of these cells are similar to those found in renal angiomyolipomas, suggesting a common origin. The distribution of the lesions, which are more common in the pelvis and along the axial axis of the lymph nodes, suggests an abdominal origin, and lesions containing LAM cells are also found in the uterus. The strongest evidence that LAM is a systemic disease comes from the recurrence described in patients who have undergone lung transplantation, suggesting its location in the lymphatic tissue. In that context, LAM is considered a low-grade neoplasm with metastatic potential.^2^ ^,^ ^16^ ^,^ ^17^

Lymphangiogenesis is essential in LAM and is involved in the chylous manifestations of the disease. It is believed to be mediated by the secretion of factors such as VEGF-C and VEGF-D by LAM cells. Those factors promote the proliferation and migration of lymphatic endothelial cells, as well as facilitating the migration of LAM cells.^16^

Estrogen appears to be closely related to LAM, given that it is a condition that is practically exclusive to women, affecting them mainly during menacme, and that there are receptors for this hormone in LAM cells.^18^ ^,^ ^19^ In addition, pregnancy, hormone replacement therapy, and infertility treatment, situations in which there is increased exposure to estrogen, have been associated with the onset and worsening of the disease.^20^

Functionally, most patients with LAM present obstructive disorder, air trapping and dynamic hyperinflation during exercise, mainly related to cystic destruction of the lung parenchyma due to an imbalance between metalloproteinases (MMPs) and their inhibitors, as well as cell proliferation and direct involvement of the small airways.^21^ ^,^ ^22^

## DIAGNOSIS

### Clinical aspects of pulmonary and extrapulmonary involvement

The clinical presentation of LAM is quite varied. Some patients are asymptomatic, whereas others present with insidious symptoms or with rapid progression until lung transplantation is required.^1^ ^-^ ^3^ Nonspecific clinical manifestations and normal chest X-ray findings in the initial evaluation contribute to a delayed diagnosis. On average, the time from the onset of symptoms to the diagnosis of LAM is 3-5 years, usually occurring in the third or fourth decade of life.^23^ Patients are often initially misdiagnosed as having asthma or COPD until a more detailed investigation is carried out on the basis of the lack of a response to treatment for those diseases.^2^ ^,^ ^3^

Many patients are asymptomatic, with pulmonary cysts being incidental findings during abdominal or thoracic imaging for various reasons, supporting the indication for LAM screening in those with TSC. Most patients with LAM present with insidious and often progressive dyspnea on exertion or a history of spontaneous pneumothorax (30-50%), which is often recurrent. Other possible clinical manifestations include cough, wheezing, hemoptysis/hemoptoic sputum (in 20%), chylothorax (in 10-30%), chyloptysis, chylous ascites, and chyluria.^2^ ^,^ ^3^ ^,^ ^24^

Renal angiomyolipomas, which are the most common extrapulmonary manifestations in LAM and are present in up to 50% of patients, can cause pain, increased abdominal volume, and hemorrhage, especially when larger than 4 cm in diameter.^2^ ^,^ ^3^ ^,^ ^12^ Lymphangioleiomyomas, which occur in approximately 16% of cases, can cause abdominal and pelvic pain, as well as edema of the lower limbs due to compression of the lymphatic and venous systems.^2^ ^,^ ^3^ ^,^ ^25^

Among patients with TSC,^3^ ^,^ ^5^ there can be dermatological manifestations, including Shagreen patches, facial angiofibromas, periungual fibromas, and hypomelanotic macules; neurological manifestations, including subependymal nodules, cortical tubers, giant cell astrocytomas, seizures, and cognitive deficit; ocular manifestations, including retinal hamartoma; and cardiac manifestations, including rhabdomyoma.

### Lung function

In the initial evaluation of patients with LAM, pulmonary function testing (PFT) is essential, mainly to identify the severity of lung involvement and to assist in decision-making regarding treatment and prognosis. In addition, PFT is essential in longitudinal follow-up, to monitor the symptoms and to assess the response to treatment.^2^ ^,^ ^26^ ^-^ ^28^

The main functional changes found in LAM are attributable to infiltration of the lung parenchyma by LAM cells and remodeling resulting from cysts, in addition to goblet and squamous cell hyperplasia, epithelial metaplasia, and airway wall thickening.^21^ ^,^ ^29^

Pulmonary function is variable in LAM and can be normal in up to half of all cases. Reduced DL_CO_ is the most common functional alteration in the initial evaluation and is usually the earliest such alteration, followed by static or dynamic air trapping. Reduced DL_CO_ is observed in 40-60% of cases in the initial evaluation, whereas air trapping is observed in 40-50% and obstructive disorder is observed in 30-50%.^3^ ^,^ ^12^ ^,^ ^30^ ^-^ ^34^ A positive bronchodilator response occurs in 15-30% of patients.^3^ ^,^ ^12^ ^,^ ^33^

The main parameter used for therapeutic decision-making is FEV_1_, and its rate of decline is well documented as a prognostic marker and as a marker of response to treatment in LAM.^30^ ^,^ ^33^ ^,^ ^34^ The annual decline in FEV_1_ reported in previous studies ranged from 47 mL to 135 mL. It is believed that the discrepant annual rates of functional decline are due to measurement biases and varying levels of baseline severity and disease progression in the populations evaluated.^3^ ^,^ ^35^ ^,^ ^36^

The annual rate of decline in FEV_1_ is higher in patients with sporadic LAM, higher serum VEGF-D values, greater degree of dyspnea, greater extent of pulmonary cysts on CT, or a positive bronchodilator response.^3^ ^,^ ^30^ ^,^ ^37^ However, postmenopausal women with LAM have higher baseline FEV_1_ and DL_CO_, as well as less functional decline.^20^ The only effective medications to stabilize or reduce functional decline in LAM are mTOR inhibitors.^20^ ^,^ ^38^

### Pulmonary and extrapulmonary imaging

**Figure 1:**
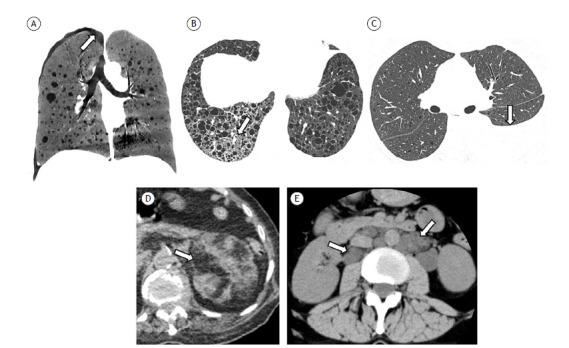
Pulmonary and extrapulmonary findings associated with lymphangioleiomyomatosis (LAM). A) Minimum-intensity projection reconstruction of a coronal CT scan, showing multiple scattered lung cysts of varying sizes, together with a small pneumothorax on the right (arrow). B) Axial CT scan of the chest, with lung window settings, showing diffuse cysts typical of LAM, together with a ground-glass opacity in the lower right lobe, presumably due to lymphatic congestion/filling or alveolar hemorrhage. C) Axial CT scan of the chest, with lung window settings, showing moderate pleural effusion, proven to be chylous in the laboratory analysis. D) Unenhanced axial CT scan of the abdomen, showing multiple nodular lesions with adipose attenuation in the renal parenchyma, characteristic of angiomyolipomas. E) Axial CT scan of the abdomen showing multiple retroperitoneal hypoattenuating nodular lesions, characteristic of lymphangioleiomyomas.

As illustrated in Figure 1, the characteristic (diagnostic) pattern of LAM on CT is that of multiple round, uniform, thin-walled, diffusely distributed pulmonary cysts.^2^ ^,^ ^28^ In the algorithm suggested in the guidelines of the American Thoracic Society and the Japanese Respiratory Society, the second step in the diagnostic workup of LAM, after clinical evaluation, is HRCT, and the identification of the classic CT pattern defines the diagnosis if associated with other findings, such as angiomyolipomas and lymphangioleiomyomas, the presence of TSC or elevated serum levels of VEGF-D.^28^ Chest X-ray might not demonstrate pulmonary alterations early in the disease and can show minimal reticulation in some patients in the advanced stages of the disease.^2^

**Figure 2:**
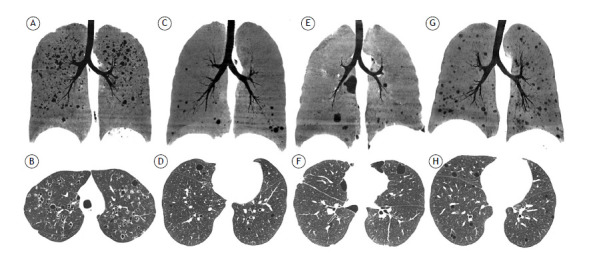
Differential diagnosis of lymphangioleiomyomatosis. Minimum-intensity projection reconstructions of coronal CT scans in A, C, E and G, and axial CT scans, with lung window settings, in B, D, F and H. In A and B, pulmonary Langerhans cell histiocytosis, with cysts of variable sizes/shapes and some small centrilobular nodules, predominantly in the upper lung fields. Note that the costophrenic recesses are preserved. In C and D, lymphocytic interstitial pneumonia, with some of the cysts having an axial distribution, mainly peribronchovascular, concentrated in the lung bases. In E and F, Birt-Hogg-Dubé syndrome, in which the cysts are typically larger and elliptical, usually with a paramediastinal distribution in the lower lung fields. In G and H, an unusual manifestation of constrictive bronchiolitis, with sparse, randomly distributed cysts.

Although HRCT has some specificity in recognizing the pulmonary manifestation of LAM, the method alone is not recommended for definitive diagnosis in patients without additional confirmatory features.^28^ The differential diagnosis of LAM on HRCT (Figure 2) includes emphysema, bronchiectasis, and honeycombing, together with other diffuse cystic lung diseases, such as Langerhans cell histiocytosis, Birt-Hogg-Dubé syndrome, lymphocytic interstitial pneumonia, and bronchiolitis.^39^ ^,^ ^40^ A small number of cysts can be observed as a consequence of lung aging. It has been suggested that a minimum of 4 would be sufficient to investigate cystic lung diseases, and that 4-10 cysts should be sufficient to raise the suspicion of a diagnosis of LAM.^2^

Other pulmonary and extrapulmonary manifestations can be seen in LAM. Ground-glass pulmonary opacities can occur and are usually secondary to smooth muscle proliferation, alveolar hemorrhage, or lymphatic congestion (Figure 1). Interlobular septal thickening, chylous pleural effusion, pericardial effusion, thoracic duct dilation, and mediastinal lymph node enlargement can also occur and represent involvement of the lymphatic compartment.^2^ Pneumothorax is common, with a high recurrence rate.^41^

Angiomyolipomas are seen in about half of all cases of LAM, in the sporadic form (in 30-40% of cases) and in LAM-TSC (in 90%). They are benign mesenchymal tumors categorized in the perivascular epithelioid cell tumor (PEComa) family and are most common in the kidneys, although they can occur at other sites, such as in the liver and lungs.^2^ ^,^ ^42^ Their most characteristic aspect is the presence of fat, which allows the definitive diagnosis to be made by imaging (Figure 1). A small proportion of these tumors can be low in adiposity, which should prompt the differential diagnosis with other neoplasms.

**Figure 3:**
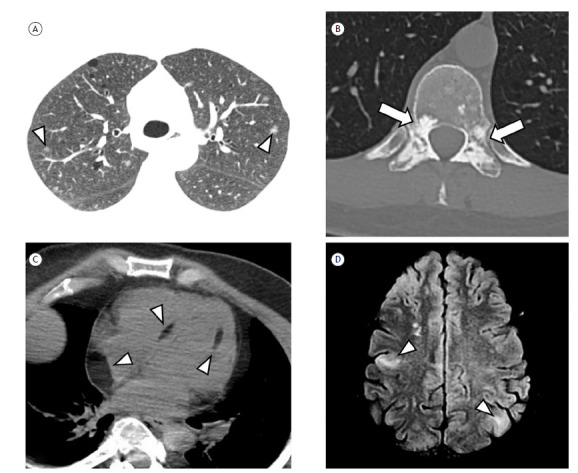
Other lesions associated with tuberous sclerosis complex. A) Axial CT scan of the chest, showing scattered pulmonary cysts related to lymphangioleiomyomatosis and some ground-glass micronodules, presumably associated with micronodular and multifocal hyperplasia of type II pneumocytes (arrowheads). B) Axial CT scan of the dorsal spine, showing multiple sclerotic foci concentrated in the posterior vertebral elements (arrows). C) Axial CT scan of the chest, with mediastinal window settings, showing foci of myocardial fat accumulation (arrowheads). D) Axial MRI of the skull, with fluid-attenuated inversion recovery weighting, showing cortical tubers (arrowheads).

In the context of LAM-TSC (Figure 3), other lesions can be identified in multiple systems, such as the central nervous system (multiple cortical tubers, subependymal nodules, and subependymal giant cell astrocytoma), heart (rhabdomyoma), kidneys (cysts and angiomyolipomas), and musculoskeletal system (sclerotic bone lesions). In the lung parenchyma, there can be micronodular and multifocal hyperplasia of type II pneumocytes, which present as multiple solid or ground-glass micronodules, measuring 2-14 mm, with a random distribution.^5^ ^,^ ^43^ There can also be well-defined foci of myocardial fat, usually in the interventricular septum or in the walls of the left ventricle.^42^

**Figure 4:**
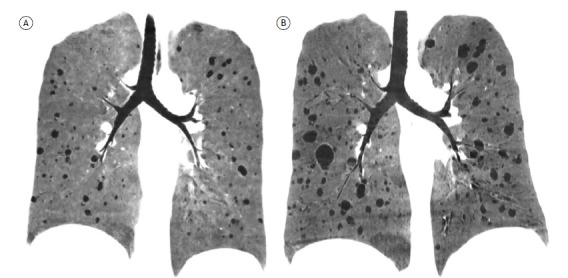
Evolution of the cystic pulmonary manifestation of lymphangioleiomyomatosis. Coronal CT scans of the chest, with minimum-intensity projection reconstruction, obtained at baseline (A) and at five years after diagnosis (B), showing progression of the condition, with increases in the number and size of the cysts.

Chest CT also plays an important role in the staging and monitoring of LAM (Figure 4). Semi-automated and automated quantification methods can be used for staging and the monitoring of progression, with the assessment of cyst extension, showing a good correlation with lung function.^44^ ^-^ ^49^ Because the longitudinal assessment of LAM can require repeated CT examinations, radiation exposure is a relevant concern, especially in young patients and female patients of reproductive age.^41^ In this context, CT scans using low-dose and ultra-low-dose radiation protocols have shown results comparable to those obtained with conventional doses for monitoring the progression of lung cysts.^46^ ^,^ ^47^

### Serum level of VEGF-D

The glycoprotein VEGF-D, which is produced by LAM cells, has been extensively studied as a biomarker of LAM. In cases of diagnostic uncertainty, the measurement of VEGF-D is particularly useful-in the context of investigation of the etiology of diffuse cystic lung disease in various populations, including that of Brazil, a serum VEGF-D level above 800 pg/mL has a specificity of nearly 100% for the diagnosis of LAM, with the potential to preclude the need for lung biopsy in patients without other clinical manifestations.

In LAM, the serum VEGF-D level correlates with the severity of lung disease and chylous manifestations, being significantly reduced after treatment with sirolimus.^28^ ^,^ ^50^ ^,^ ^51^ However, there are a number of limitations to its use. There is great variability across studies in terms of its accuracy and ideal cutoff value, which ranges from 440 pg/mL to over 1,200 pg/mL. In addition, it has moderate sensitivity, and its levels are higher in patients with LAM-TSC, lymphatic involvement, and extrapulmonary manifestations, in which its measurement could be considered unnecessary. Furthermore, it has no confirmed clinical prognostic value; nor have there been any studies showing its usefulness in monitoring disease activity during therapy.^51^ ^-^ ^53^ Moreover, this test is still not widely available in Brazil.

Mainly due to its potential to preclude the need for invasive procedures, VEGF-D measurement, despite its limitations, is still recommended in the investigation of patients with suspected LAM and without other manifestations that could confirm the diagnosis.^2^ ^,^ ^26^ ^,^ ^28^

### Biopsy and histopathological aspects

Lung biopsy can be performed to confirm the diagnosis of LAM when the results of the clinical examination, CT evaluation, and measurement of the serum VEGF-D level are not sufficient to reach a conclusion and when the benefits of a biopsy outweigh the risks of the procedure.^2^ ^,^ ^26^ In patients with diffuse, asymptomatic cysts and normal or slightly altered lung function, periodic monitoring alone can be used, without a need for biopsy.^2^ Transbronchial lung biopsy has a sensitivity of over 50% and can be used as the initial invasive method at centers with experience in its use.^28^ ^,^ ^54^ ^,^ ^55^ Transbronchial cryobiopsy can also be an option, as demonstrated in specific cases.^56^ ^,^ ^57^ When there is uncertainty about performing transbronchial biopsy or when the results of such a biopsy are inconclusive, surgical lung biopsy is recommended, preferably by video-assisted thoracoscopy.^26^ ^,^ ^27^

The characteristics of LAM include abnormal proliferation of cells expressing smooth muscle proteins in the lungs, axial lymph nodes, and other sites, often accompanied by renal angiomyolipoma.^58^ The disease is classified by the World Health Organization in the group of PEComas, characterized as mesenchymal tumors composed of histologically and immunohistochemically distinct perivascular epithelioid cells.^59^ The proliferation of LAM cells appears to play a central role in the destruction of the lung parenchyma.^60^

**Figure 5:**
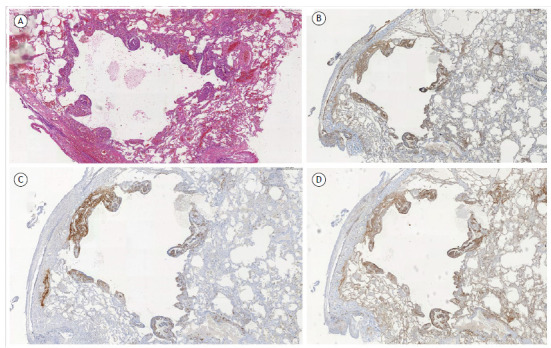
Photomicrographs demonstrating histopathological and immunohistochemical aspects in a patient with lymphangioleiomyomatosis (LAM). A) Proliferation of LAM cells with formation of small nodules throughout a pulmonary cystic lesion (H&E). B) Immunohistochemistry showing LAM cells that stained positive for smooth muscle actin (B); HMB-45 (C); and beta-catenin (D, membrane and cytoplasmic patterns).

Lesions in LAM are composed of two cell subpopulations: spindle-shaped, myofibroblast-like cells; and polygonal cells with an epithelioid morphology. LAM cells predominantly form nodules (Figure 5A), although small cell clusters can be found scattered throughout the lung parenchyma.^61^ Spindle-shaped cells express specific smooth muscle proteins, such as smooth muscle actin (Figure 5B), desmin, and vimentin, and form the core of the nodule, surrounded by epithelioid cells that exhibit immunoreactivity for the HMB-45 antibody (Figure 5C), which binds to the glycoprotein gp100, a marker of melanocytes.^62^ Spindle-shaped cells appear to represent a component with greater proliferative activity and are more closely related to the destruction of lung connective tissue due to the release of MMPs.^63^ ^,^ ^64^ Spindle-shaped cells show abundant staining for MMPs, mainly MMP-2, MMP-9, and MT1-MMP.^60^ ^,^ ^64^

The gold standard marker for the diagnosis of LAM is HMB-45, which has high specificity but has variable sensitivity when the biopsy specimen is small.^55^ ^,^ ^65^ ^,^ ^66^ Beta-catenin (Figure 5D) can be a useful marker due to its high sensitivity, with labeling of both cell subtypes, and high specificity, because it is not expressed in the smooth muscle of airway or vascular walls.^67^ Cathepsin K, a papain-like cysteine protease with matrix degradation activity, appears to be more sensitive than is HMB-45 for the diagnosis of the disease.^66^ In addition, LAM cells express estrogen and progesterone hormone receptors.^18^ ^,^ ^68^ The role of estrogen in disease progression is not yet fully established, although there is evidence that it signals through AKT.^62^

The cause of TSC is a germline mutation in the *TSC1* gene or *TSC2* gene, located on chromosomes 9q34 and 16p13, respectively.^63^ ^,^ ^69^ ^,^ ^70^ Acquired mutations in those genes are likely the cause of sporadic LAM, with mutations occurring more frequently in *TSC2* than in *TSC1*. Both are tumor suppressor genes, and loss of heterozygosity for *TSC2* has been reported in LAM lesions of the lung and kidney.^71^

The tumor suppressor genes *TSC1* and *TSC2* encode the proteins hamartin and tuberin, respectively. The phenotypic and symptomatic similarities between patients carrying *TSC1* mutations and those carrying *TSC2* mutations suggest that the functions of hamartin and tuberin are intertwined in the cellular signaling pathway.^69^ Characterization of the *TSC1* and *TSC2* genes has allowed functional studies that have led to the current understanding of the signaling pathways for hamartin and tuberin.^63^ The hamartin-tuberin complex acts as a GTPase-activating protein against Rheb (a Ras homologue enriched in the brain), which regulates mTOR signaling. Phosphorylation and activation of the p70 ribosomal protein S6 kinase by mTOR leads to activation of the ribosomal protein S6 via phosphorylation at Ser240\244. The mTOR signaling pathway plays a central role in regulating cell growth in response to growth factors, cellular energy, and nutrient levels.^72^ ^,^ ^73^ The hamartin-tuberin complex negatively regulates Rheb by converting Rheb-GTP to Rheb-GDP, thus inactivating Rheb and inhibiting mTOR.^74^ Therefore, dysfunction in the encoding of these proteins results in dysregulation of signals, such as those related to cell surface receptor tyrosine kinase and G-protein-coupled receptor. Constitutive activation of mTOR kinase and S6 kinase leads to increased protein translation, with inappropriate cell proliferation, migration, and invasion.^75^

### Six-minute walk test and cardiopulmonary exercise test

Reduced exercise tolerance is common in LAM, and examinations performed at rest might not reveal any alterations; in fact, many patients have normal PFT results. In this context, it is important that patients be evaluated during exercise-based examinations, such as the six-minute walk test (6MWT) and incremental cardiopulmonary exercise testing (CPET).^31^ ^,^ ^76^

The 6MWT is a submaximal test, and the main parameter evaluated is the six-minute walk distance (6MWD). Although it is an important test for assessment of the severity and progression of LAM, the result is often normal, even in patients with functional limitations. A study conducted in Brazil demonstrated that patients with LAM walked approximately 90% of the predicted 6MWD, but 35% of those patients showed ≥ 4% desaturation.^77^

One recent study of patients with LAM evaluated the desaturation-distance ratio (DDR), an index calculated from the ratio between the desaturation area and the 6MWD or the distance walked on the shuttle walk test with Holter oximetry, which has been correlated with a reduction in FEV_1_, a reduction in DL_CO_, and air trapping. The DDR shows promise in the functional assessment of LAM, because it expands the analysis of isolated parameters of the 6MWT.^78^

Although CPET provides a more comprehensive and maximal assessment, with analysis of metabolic, ventilatory, and cardiovascular variables, it is less widely available and more expensive. It is indicated when there is uncertainty about the cause of dyspnea on exertion and is important to help define training parameters in pulmonary rehabilitation.^31^ ^,^ ^77^ Reduced exercise capacity and maximal oxygen consumption are common findings in LAM, especially in patients with more advanced disease.^76^ ^-^ ^78^ The mechanisms of exercise limitation in LAM are often multifactorial, including ventilatory limitation, dynamic hyperinflation, reduced gas exchange, pulmonary hypertension (PH), and peripheral muscle fatigue.^31^ ^,^ ^76^

### Echocardiogram and PH

Echocardiography is an essential tool in the evaluation of various pulmonary conditions and can aid in the management of the disease, especially when PH is suspected. The main objectives of echocardiography in LAM include the evaluation of the heart chambers and the occurrence of PH, a possible complication of the disease.^79^ ^,^ ^80^

Changes in the lung parenchyma associated with LAM can lead to PH, that may determine right ventricular overload, which can lead to heart failure and worsening of dyspnea. The PH associated with LAM is usually classified as group III (resulting from parenchymal disease or hypoxemia), has a low (≤ 10%) prevalence, and is usually mild in intensity.^79^ ^,^ ^81^ ^,^ ^82^ A reduction in DL_CO_ increases the sensitivity for predicting the occurrence of PH, especially when it is ≤ 40% of predicted.^79^ When echocardiography is combined with the assessment of DL_CO_, invasive hemodynamic assessment becomes increasingly less indicated in LAM.^79^ ^,^ ^80^ Other parameters can be evaluated to raise the suspicion of PH in LAM, such as the ratio between the diameter of the pulmonary artery and that of the aorta on chest CT.^83^

Although PH at rest is rare in LAM, an increase in pulmonary artery pressure at low levels of exertion occurs more frequently, affecting up to 60% of patients.^81^ Exercise-induced PH in LAM is believed to be related not only to hypoxic pulmonary vasoconstriction (precapillary PH) but also to a significant increase in pulmonary capillary wedge pressure, probably secondary to diastolic dysfunction (postcapillary PH).^84^ Therefore, the effects that LAM has on pulmonary function could also have repercussions for cardiac involvement, and echocardiography can provide additional information for the overall assessment of the impact of the disease. Identifying the relationship between pulmonary changes and cardiac complications could aid in the management of the disease and in optimizing patient quality of life. The frequency of echocardiography in LAM should be individualized, and there is still no consistent evidence for the specific treatment of PH in patients with the disease.

### Diagnostic algorithm

**Figure 6:**
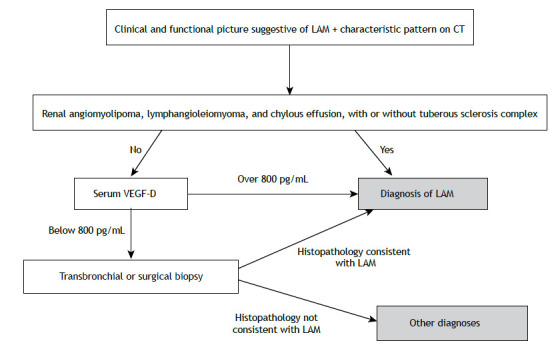
Algorithm for the diagnostic approach to lymphangioleiomyomatosis (LAM ).

Chart 1 presents the main clinical characteristics and ancillary examinations. Figure 6 shows the algorithm for the diagnostic approach to LAM.

**Chart 1:** Main characteristics of lymphangioleiomyomatosis seen on clinical examinations and ancillary tests.

Clinical characteristics that can be seen over the course of the disease	- Asymptomatic - Progressive dyspnea on exertion - Pneumothorax - Cough, wheezing - Hemoptysis, hemoptoic sputum - Chylothorax, chyloptysis - Chylous ascites, chyluria - Renal angiomyolipoma - Abdominal and pelvic lymphangioleiomyomas - Dermatological manifestations, such as Shagreen patches, facial angiofibromas, periungual fibromas, and hypomelanotic macules - Neurological manifestations, such as subependymal nodules, cortical tubers, giant cell astrocytomas, seizures, and cognitive impairment - Retinal hamartoma - Cardiac rhabdomyoma
Lung function	- Normal (in up to 50% of cases) - Reduction of DL_CO_ (in 40-60%) - Air trapping (in 40-50%) - Obstructive ventilatory disorder (in 30-50%) - Positive response to bronchodilator (in 15-30%)
Imaging	Chest CT - Diffuse, regular, thin-walled pulmonary cysts - Pneumothorax, chylous pleural effusion - Areas of ground-glass opacity, interlobular septal thickening - Dilation of the thoracic duct - Mediastinal lymph nodes enlargement - Micronodules (multifocal and multinodular hyperplasia of pneumocytes) - Sclerotic bone lesions - Relationship between the diameter of the pulmonary artery and the aorta can increase CT/MRI of abdomen and pelvis - Renal and hepatic angiomyolipomas - Lymphangioleiomyomas CT/MRI of the skull - Cortical tubers - Subependymal astrocytoma - Subependymal nodules
Serum VEGF-D	- Can be normal - > 800 pg/mL (high specificity)
Histopathological features	- Spindle cells (SMA, beta-catenin, MMPs) - Epithelioid cells (HMB-45, beta-catenin) - Formation of nodules - Lung cysts
Six-minute walk test	- Reduced distance covered - Desaturation above 4%
Cardiopulmonary exercise test	- Reduced maximum oxygen consumption - Multifactorial limitation (ventilatory, dynamic hyperinflation, altered gas exchange, PH and peripheral muscle)
Echocardiogram	- Mainly group III PH - Mild PH (in < 10% of cases)

SMA: smooth muscle actin; PH: pulmonary hypertension; and MMPs: metalloproteinases.

## TREATMENT

The indications for the use of mTOR inhibitors and a summary of the main therapeutic measures in LAM are presented in Charts 2 and 3, respectively.

**Chart 2:** Main indications for the use of mTOR inhibitors in lymphangioleiomyomatosis.

Pulmonary involvement	- FEV_1_ ≤ 70% predicted - Annual drop in FEV_1_ ≥ 90 mL - Hypoxemia at rest or on exertion - Recurrent pneumothorax*
Extrapulmonary manifestations	- Renal angiomyolipomas ≥ 4 cm diameter - Symptomatic abdominal and pelvic lymphangioleiomyomas - Symptomatic chylous effusions

*Although the benefits are not yet fully established, mTOR inhibitor use can be considered if there is recurrent pneumothorax.

**Chart 3:** Other therapeutic approaches in lymphangioleiomyomatosis.

Inhaled bronchodilators	- Symptomatic patients with obstructed lung function, especially if they show a positive bronchodilator response
Pneumothorax	- Treat after the initial episode due to the high risk of recurrence - Pleurodesis with talc, mechanical abrasion or pleurectomy - Trend toward indication of mTOR inhibitors for recurrent pneumothorax
Chylothorax	- mTOR inhibitors for symptomatic and persistent cases - A low-fat diet rich in medium-chain triglycerides or pleural drainage might be necessary, temporarily - Ligation or embolization of the thoracic duct with or without pleurodesis for refractory cases
Renal angiomyolipoma	- mTOR inhibitors, if ≥ 4 cm diameter or symptomatic - Arterial embolization for bleeding or if there are microaneurysms ≥ 5 mm in diameter - Partial nephrectomy for refractory cases or if kidney cancer is suspected or confirmed
Lymphangioleiomyoma	- mTOR inhibitors, if symptoms occur
Rehabilitation and physical exercise	- Rehabilitation with monitoring for patients with severe functional impairment, desaturation, cardiovascular risk, or fall risk - Recommend physical activity, even outside of the rehabilitation regimen, for those without severe limitations and without contraindications - Ideal: aerobic and strength exercise - No increase in effort-related adverse events
Supplemental oxygen	- PaO_2_ ≤ 55 mmHg at rest - PaO_2_ of 56-59 mmHg at rest, if PH, edema due to heart failure, or hematocrit above 55% - During exertion and sleep, if hypoxemia occurs
Lung transplant	- FEV_1_ < 30% of predicted; hypoxemia at rest; New York Heart Association functional class III or IV; or progressive functional loss despite optimized treatment

PH: pulmonary hypertension; and mTOR: mechanistic target of rapamycin.

### mTOR inhibitors

The main medications used in the treatment of LAM are mTOR inhibitors, especially sirolimus.^26^ A randomized, placebo-controlled trial of patients with LAM who had an FEV_1_ ≤ 70% of predicted demonstrated that a 12-month course of sirolimus slowed the decline in lung function, improved patient quality of life, and reduced serum VEGF-D levels.^38^ ^,^ ^85^ The patients were followed for 12 months after discontinuation of the medication, during which period there was resumption of the decline in lung function.^38^ Other studies have demonstrated the benefit of sirolimus in LAM, in terms of its effects on functional loss and involvement of the lung parenchyma on chest CT, as well as a reduction in mortality.^37^ ^,^ ^44^ ^,^ ^86^ ^-^ ^89^

Sirolimus is indicated for pulmonary involvement in LAM when the FEV_1_ is < 70% of predicted, when the annual decline in FEV_1_ is ≥ 90 mL, or when there is hypoxemia (at rest or on exertion).^2^ ^,^ ^26^ ^,^ ^27^ Its benefits extend to premenopausal and menopausal women alike.^20^ Sirolimus is highly effective in improving extrapulmonary manifestations, such as renal angiomyolipomas, lymphangioleiomyomas, and chylous effusions. For renal angiomyolipomas, the drug is indicated when the tumor is > 4 cm in diameter.^25^ ^-^ ^27^ For chylous effusions, it is recommended that mTOR inhibitors be used before invasive procedures are indicated.^26^

Although recurrent pneumothorax is not yet a definitive indication for mTOR inhibitors, sirolimus appears to reduce the risk of it.^90^ ^,^ ^91^ Therefore, its use can be considered for cases of recurrent pneumothorax in LAM.

Sirolimus is generally well tolerated, and adverse effects are mild, occurring mainly in the first six months of use.^26^ ^,^ ^92^ The serum level of the drug should be monitored, and the recommendation is that it be maintained between 5 ng/mL and 15 ng/mL. The most common adverse events are mucositis, diarrhea, abdominal pain, nausea, hypercholesterolemia, hyperglycemia, acne, upper respiratory tract infections, menstrual changes, lower limb edema, anemia, lymphopenia, and thrombocytopenia.^2^ ^,^ ^26^ ^,^ ^38^

The ideal initial and maintenance doses of sirolimus have yet to be fully established.^2^ It has been shown that even in patients with serum levels < 5 ng/mL, there can be improvement or stabilization of lung function and resolution of chylous effusions, suggesting that in these situations, the initial dose can be lower, such as 1 mg/day.^86^ ^,^ ^89^ We recommend an initial oral sirolimus dose of 1-2 mg/day, with serum levels measured two weeks after initiation, then monthly for three months, and every three months thereafter.

It should be borne in mind that sirolimus is not a curative or definitive treatment and should be given continuously and indefinitely or at least until the onset of menopause, when the progression of the condition can be assessed from the hormonal decline.^26^ ^,^ ^38^ Various studies have shown that sirolimus is safe and effective in the long term, with low rates of discontinuation and serious adverse events, as well as having beneficial effects on lung function, exercise capacity, quality of life, renal angiomyolipoma, lymphangioleiomyomas, chylous effusions, and serum VEGF-D.^92^ ^-^ ^94^ Long-term benefits have been demonstrated for premenopausal and menopausal women alike.^94^ Long-term adverse events are similar to those described during the first year of treatment.^92^ ^-^ ^94^

Only a few studies have evaluated the use of everolimus in LAM, demonstrating benefits on functional decline and exercise capacity, with an adverse event profile similar to that of sirolimus.^95^ Everolimus can be considered an alternative in cases of intolerance of or refractoriness to sirolimus.

Sirolimus is approved for the treatment of LAM in Brazil. Details regarding the use of mTOR inhibitors in the context of lung transplantation and complications are described below, in specific items.

### Inhaled bronchodilators

Inhaled bronchodilators can be used in symptomatic patients with airway obstruction, especially if there is a positive bronchodilator response, and should be continued if there is clinical improvement.^27^ ^,^ ^33^ ^,^ ^96^ ^-^ ^98^ Inhaled corticosteroids are not recommended in the management of LAM.^27^

### Approach to pneumothorax

Pneumothorax is a common complication of LAM; 30-50% of patients will experience it during the course of the disease.^3^ ^,^ ^12^ ^,^ ^99^ ^-^ ^101^ The reported risk of pneumothorax recurrence is high, reaching 70%.^19^ ^,^ ^99^ ^,^ ^101^ One prospective study demonstrated that there was no relationship between the occurrence/number of pneumothorax events and functional decline, progression to death, or the need for lung transplantation.^30^

Patients with LAM should be informed of the increased risk of pneumothorax and how to recognize its signs and symptoms, facilitating its early detection. A retrospective study demonstrated that the incidence of pneumothorax was higher in women with LAM than in the general female population and that the risk of developing pneumothorax was three times higher after air transport.^102^

Immediate, definitive treatment of pneumothorax is recommended after the initial episode.^28^ ^,^ ^30^ It should be emphasized that prior pleurodesis is not a contraindication for lung transplantation, supporting its indication after the initial pneumothorax.^2^ ^,^ ^28^ There is still no consensus regarding the ideal method of pleurodesis in LAM. Talc pleurodesis is the technique of choice at most referral centers in Brazil. However, pleurodesis by mechanical abrasion or pleurectomy can also be performed but, in Brazil, is generally reserved for cases of recurrent pneumothorax or pneumothorax refractory to other procedures.^3^ It is recommended that patients avoid air travel for at least four weeks after undergoing a pleural procedure.^102^

Recently, an alternative to pleurodesis, with pleural coverage of the entire visceral surface of the lung by video-assisted thoracoscopy, was described as a treatment for LAM, effectively reducing the recurrence of pneumothorax without causing ventilatory impairment or pleural adhesion. However, this technique is still limited to a few centers.^103^

Inhibitors of mTOR appear to reduce the risk of pneumothorax recurrence, and it is recommended that they be discontinued for at least one week before and two to four weeks after pleurodesis, to allow adequate healing.^90^ ^,^ ^91^ ^,^ ^104^ Therefore, although it is not yet considered a definitive indication in LAM, the use of sirolimus can be recommended for cases of recurrent pneumothorax, whether or not the case meets the other criteria for its use.

### Approach to chylothorax

In LAM, chylothorax, which occurs in 10-30% of cases, is caused by rupture or blockage of the thoracic duct or one of its branches by LAM cells or by transdiaphragmatic flow of chylous ascites, mainly being unilateral (in the right hemithorax).^3^ ^,^ ^105^ ^,^ ^106^

In LAM, effusions have a variable clinical course and can remain stable over time. Periodic monitoring, with or without thoracentesis, is usually sufficient for small, asymptomatic chylothorax.^106^ In cases of symptomatic, persistent chylothorax, treatment with sirolimus is indicated and patients typically respond well. Chylothorax can take up to a year to resolve after the start of treatment with sirolimus, often requiring additional therapy with a low-fat diet rich in medium-chain triglycerides or with pleural drainage until a consistent effect of the drug is achieved.^25^ ^,^ ^107^ If the patient already begins or continues to have high chylothorax output after the initial measures, fasting and total parenteral nutrition can be initiated.^108^ ^,^ ^109^

An invasive approach should be considered only after treatment with sirolimus has been attempted.^87^ ^,^ ^107^ In patients with sirolimus-resistant chylous complications or with contraindications to sirolimus, surgical ligation of the thoracic duct, with or without pleurodesis, is suggested. Pleurodesis can be performed by talc abrasion.^110^ Although percutaneous interventional radiology techniques are available to embolize the duct in chylothorax, there have been no studies showing consistent results in LAM.^111^ Scintigraphy or MRI of the lymphatic vasculature and transfer to a referral center for LAM are recommended if surgical or percutaneous management of chylothorax is necessary. For sirolimus-resistant cases, switching to everolimus can also be considered.

### Approach to renal angiomyolipoma

Angiomyolipomas are common in LAM and are characterized as benign tumors of mesenchymal origin, rich in fat, muscle tissue, and blood vessels; they can be found in the kidneys, liver, intestine, and bladder.^2^ ^,^ ^3^ ^,^ ^12^ Although renal angiomyolipomas are generally small, mostly unilateral and asymptomatic, they can evolve to hemorrhage and a high risk of death, especially if they are > 4 cm in diameter or have aneurysmal vascularization.^2^ They are usually asymptomatic, although there can be mild abdominal pain, hemorrhage, and renal failure (requiring dialysis and kidney transplantation).^2^ ^,^ ^19^

Few renal angiomyolipomas require treatment; when treatment is required, the primary goals are to prevent bleeding and preserve renal function.^27^ ^,^ ^112^ Treatment is required if there are symptoms such as abdominal pain and vomiting, as well as if the tumor is ≥ 4 cm in diameter, if there is exophytic growth, if there are microaneurysms ≥ 5 mm in diameter, or if the tumor is highly vascularized, all of which increase the risk of bleeding.^113^ There are three therapeutic options: mTOR inhibitors, arterial embolization by catheterization, and tumor resection surgery.^2^ ^,^ ^113^

Arterial embolization can reduce the tumor volume by up to 80%, although there is a risk of recurrence and kidney damage due to the procedure, which is therefore reserved for cases of bleeding or for embolization of intratumoral microaneurysms with a diameter ≥ 5 mm.^2^ ^,^ ^113^ ^-^ ^115^

For the treatment of renal angiomyolipoma, mTOR inhibitors are highly effective.^26^ A phase II trial evaluated 20 patients with renal angiomyolipoma and found a 53% reduction in lesion volume after 12 months of treatment with sirolimus, with an increase in tumor volume after the drug was discontinued.^116^ Two other studies demonstrated similar results.^117^ ^,^ ^118^ A double-blind, randomized clinical trial evaluated the use of 10 mg/day of everolimus in patients with renal angiomyolipoma.^112^ After six months of treatment, 55% of the patients in the everolimus group showed a reduction of at least 50% in tumor volume and 80% of those patients showed a reduction of at least 30% in total volume, with this effect increasing after two years of use.^112^ ^,^ ^119^ ^,^ ^120^

The efficacy and safety of mTOR pathway inhibitors have made them the standard treatment for renal angiomyolipomas associated with TSC or sporadic LAM. However, the ideal duration of treatment has not been established, and treatment should be continued indefinitely as long as there is a clinical and radiological response.^2^ ^,^ ^113^ In cases of drug intolerance, an intermittent regimen can be tried, with the medication being discontinued when the tumor has shrunk to a diameter < 4.0 cm and resumed when new tumor growth occurs.^121^

Nephrectomy, usually partial, is indicated only in rare cases, especially those in which the response to other treatments is inadequate or kidney cancer is suspected or confirmed.^27^

### Approach to lymphangioleiomyomas

Infiltration of lymphatic tissue by LAM cells can cause lymph node enlargement, chylous effusions, and lymphangioleiomyomas (in up to 16% of cases), especially in the abdominal and pelvic cavities.^2^ ^,^ ^3^ Biopsy and surgical resection of lymphangioleiomyomas should be avoided. To our knowledge, there have been no randomized clinical trials evaluating treatment with mTOR inhibitors in patients with lymphangioleiomyomas. However, case series have shown good clinical and radiological responses after the use of these medications, with a significant reduction or disappearance of the lesion after six months of treatment.^25^ ^,^ ^122^ ^,^ ^123^ The effect of the drug appears to have an early onset, often two weeks after the start of treatment. Asymptomatic lesions can simply be monitored, without the need for treatment. Inhibitors of mTOR are indicated for symptomatic cases, especially those in which there is abdominal discomfort or pain, and should be continued at least until the lesions in question resolve.^123^

### Rehabilitation and physical exercise

Pulmonary rehabilitation, including aerobic and strength exercises, improves exercise capacity in LAM, as demonstrated by increased endurance/improved metabolic variables on constant-load CPET, increased 6MWD,^77^ ^,^ ^124^ ^,^ ^125^ and better quality of life.^77^ ^,^ ^124^ One study demonstrated that yoga practice increases the 6MWD and the maximum load in the maximum CPET in LAM.^126^ Among studies of patients with LAM who engage in guided physical exercise, no increased risk of adverse events, such as pneumothorax, was observed during the exercise.^31^ ^,^ ^77^ ^,^ ^78^ Pulmonary rehabilitation should be considered for all patients with LAM who have limited physical activity, supporting the need for physician indication and monitoring.^77^

A remote rehabilitation program based on cell phone-guided exercises with heart rate and SpO_2_ monitoring demonstrated safety and increased 6MWD.^125^ Although evidence is limited, it is recommended that physical activity be encouraged, even outside of a formal rehabilitation program, for patients who, after a comprehensive medical evaluation, do not exhibit severe functional impairment, significant desaturation on exertion, significant cardiovascular risk, or a risk of falling.^77^ ^,^ ^127^ Considering the current evidence and the pathophysiology of LAM, greater emphasis on aerobic exercise is suggested.^128^ Patients with pneumothorax should wait at least four weeks after resolution of the condition to start physical activity.^127^

### Hormonal blockade

In patients with LAM, various therapies for hormonal blockade have been proposed and evaluated, including bilateral oophorectomy, as well as the use of gonadotropin-releasing hormone agonists, aromatase inhibitors, tamoxifen, and progesterone, although none of them have produced consistent results.^2^ ^,^ ^129^ ^-^ ^133^ As presented in international guidelines, hormonal blockade is not recommended for the treatment of LAM, despite very low-quality evidence to support or discourage it. Additional studies are needed in order to evaluate the role of hormonal blockade in combination with mTOR inhibitors for the treatment of the disease. It should be borne in mind that hormonal methods, especially those employing progesterone alone, can be used for contraceptive purposes.^26^

### Oxygen therapy

The recommendation for long-term oxygen therapy in LAM is extrapolated from information obtained from research in patients with severe COPD, because, to our knowledge, there have been no studies evaluating the benefits of supplemental oxygen in this disease.

Supplemental oxygen is recommended for patients with a PaO_2_ ≤ 55 mmHg on room air at rest and for those with a PaO_2_ of 56-59 mmHg who also have PH, edema due to heart failure, or hematocrit above 55%. Treatment aims to maintain SpO_2_ above 90% and should be carried out for ≥ 15 h/day, including the sleep period.^134^ ^,^ ^135^ Supplemental oxygen should be considered when there is hypoxemia during exertion or during sleep.^134^

### Lung transplantation

Patients with LAM should be referred for lung transplant evaluation when the disease is advanced, with end-stage respiratory failure, characterized by an FEV_1_ below 30% of predicted, resting hypoxemia, a New York Heart Association functional class III or IV, or progressive functional loss despite treatment.^2^ ^,^ ^27^ ^,^ ^136^ ^,^ ^137^

Patients with LAM who undergo lung transplantation have similar or better outcomes than do patients with other lung diseases, possibly because they are typically younger and usually have fewer comorbidities.^2^ ^,^ ^138^ ^,^ ^139^ Studies of patients with LAM in various regions of the world, including Brazil, have demonstrated rates of survival at 1, 3, 5, and 10 years after lung transplantation of 79-94%, 73-90%, 73-77%, and 56-74%, respectively.^138^ ^-^ ^142^

Although it increases the risk of bleeding during or after the procedure, prior pleurodesis does not contraindicate lung transplantation. Bilateral transplantation is recommended.^27^ ^,^ ^137^ It should also be borne in mind that recurrence of LAM after lung transplantation is rare and usually has no clinical or functional repercussions.^139^ ^,^ ^140^ ^,^ ^143^

For patients on the lung transplant waiting list, it is recommended that mTOR inhibitors be maintained during the waiting period and discontinued immediately before the procedure.^137^ ^,^ ^138^ ^,^ ^144^ ^,^ ^145^ Reducing the dose of the medication in the pre-transplant period can be considered.^144^ Maintaining mTOR inhibitors after transplantation increases the risk of bronchial anastomotic dehiscence by interfering with healing.^2^ ^,^ ^146^ In this context, it is suggested that the medication be restarted after complete healing of the bronchial anastomosis, which typically occurs 3 months after transplantation.^138^ ^,^ ^146^ The use of mTOR inhibitors should be evaluated after transplantation in LAM, because it could be important for controlling extrapulmonary manifestations and preventing pulmonary recurrence of the disease.^141^

## OTHER RELEVANT TOPICS

Chart 4 summarizes other relevant topics regarding LAM.

**Chart 4:** Other relevant topics in the approach to lymphangioleiomyomatosis.

Vaccination	- Avoid live virus vaccines if using mTOR inhibitors - Influenza vaccine - Pneumococcal vaccine - Recombinant vaccine against herpes zoster virus (for all > 50 years of age and all using mTOR inhibitors regardless of age) - COVID-19 vaccine
Air travel	Risk of pneumothorax - The decision of whether to travel must be made on a case-by-case basis - Avoid in cases of severe lung function impairment, extensive cysts on CT, or history of recurrent pneumothorax Supplemental oxygen in flight - No need for supplemental oxygen if SpO_2_ > 95% - perform 6MWT if SpO_2_ is 92-95%; use supplemental oxygen if SpO_2_ ≤ 84% on 6MWT - If SpO_2_ < 92%, supplemental oxygen is required
Gestation	- Individualize recommendations - Periodic PFT - Can result in a worsening of pulmonary functional decline, obstetric complications, and LAM (pneumothorax, chylous effusions, and progression of renal angiomyolipoma and lymphangioleiomyomas) - No absolute contraindication if lung function is preserved or there is mild limitation without progression - mTOR inhibitors: should generally be discontinued; consider starting or maintaining in specific situations, at low doses
Contraception and hormone replacement therapy	- Avoid medications containing estrogen - Topical vaginal estrogen can be considered - Avoid infertility treatment - Possible methods: copper or progesterone intrauterine devices; progesterone, partner vasectomy and barrier devices
Osteoporosis	- Perform bone densitometry periodically - Treatment with calcium, vitamin D, and bisphosphonates - Resistance and muscle strength training

LAM: lymphangioleiomyomatosis; mTOR: mechanistic target of rapamycin; PFT: pulmonary function test; and 6MWT: six-minute walk test.

### Vaccination

Patients with LAM should keep their vaccinations up to date, and those taking mTOR inhibitors should not receive live virus vaccines.^2^ It is essential that the administration of vaccines be discussed with the professional who is treating the patient.

Annual immunization against the influenza virus with inactivated vaccine is recommended for all patients with LAM, as is immunization with pneumococcal vaccine. The use of recombinant vaccine against herpes zoster virus is recommended for all patients with LAM who are ≥ 50 years of age and for those taking mTOR inhibitors, regardless of age.^2^ Vaccination against COVID-19 using messenger RNA technology has been shown to be safe and effective, and a recent study of patients with LAM demonstrated that the response levels were similar between the patients who were taking sirolimus and those who were not.^147^

### Air travel

Two issues related to air travel are relevant in patients with LAM: the need for supplemental oxygen; and the potential risk of pneumothorax. Most patients can travel safely, especially if lung function is normal or only mildly impaired.^102^ ^,^ ^148^ ^-^ ^150^ Patients should not travel by air until at least four weeks after resolution of pneumothorax.^27^

It has been speculated that air travel increases the risk of pneumothorax in patients with LAM due to the rupture of subpleural cysts, induced by changes in cabin air pressure.^149^ However, there have been few studies on the safety of air travel in patients with LAM, and the answers to this question are not completely clear. The incidence of pneumothorax has been shown to be approximately 1,000 times higher in women with LAM than in the general female population, the risk has been shown to be three times higher after air travel, and chemical or surgical pleurodesis has been shown to partially reduce the risk of pneumothorax recurrence in flight.^102^ In another study of patients with LAM, using a questionnaire-based assessment of air travels, 2% were found to have had a pneumothorax during the flight.^150^ However, a retrospective study of 281 patients with LAM demonstrated that the occurrence of air travel-related pneumothorax might be more related to the high incidence of this complication in the disease than to the travel itself.^151^ Therefore, travel recommendations should be individualized. Patients with symptoms that have not been elucidated before a scheduled flight, especially dyspnea and chest pain, should not board. It is recommended that patients with reduced pulmonary reserve or with high-risk characteristics, such as large cyst extension, severe impairment of lung function, and a history of multiple pneumothoraces, seek alternative modes of travel.

Air travel can expose patients with chronic respiratory diseases, including LAM, to the effects of acute hypoxemia at altitude, with the risks of worsening symptoms and complications during the flight.^134^ These risks will be especially high in patients with LAM who already have hypoxemia, even if only mild or moderate, at ground level.^152^

Patients with chronic lung disease, including LAM, with an SpO_2_ > 95% on room air can fly without supplemental oxygen. Conversely, those with an SpO_2_ < 92% should receive supplemental oxygen during the flight. Patients with an SpO_2_ between 92% and 95% should be submitted to a 6MWT or a simulated high-altitude hypoxia test, the latter of which is not widely available.^134^ ^,^ ^152^ Patients who have an SpO_2_ ≤ 84% persistently during either of those tests will require supplemental oxygen during the flight.^134^ ^,^ ^153^ Patients requiring a flow rate > 4 L/min to correct hypoxemia should be discouraged from flying and, if they do, should use air medical transport.^134^ ^,^ ^154^

### LAM and COVID-19

During the COVID-19 pandemic, the risk of death was found to be higher among patients with interstitial lung disease and the prognosis was found to be worse among those with an FVC < 80% of the predicted value.^155^ ^,^ ^156^ A retrospective multicenter study evaluated 91 patients with LAM who reported having had COVID-19, and only one death was observed among those patients. Multivariate analysis showed that DL_CO_ was a determinant of the risk of hospitalization and of the need for supplemental oxygen. The authors concluded that LAM did not increase the risk of death or of the progression to long COVID and that the use of mTOR inhibitors did not alter the prognosis.^157^

### Gestation

The population mainly affected by LAM is that of women of reproductive age, and its pathogenesis is partly related to female hormones, especially estrogen.^158^ Pregnancy is one of the most challenging periods for patients with LAM, mainly because of the high estrogen levels, although more consistent data are needed for a better understanding.^159^ ^,^ ^160^ The diagnosis of LAM can be established during or after pregnancy. Patients often report avoiding pregnancy because of the increased risk of complications.^161^ ^,^ ^162^

There is evidence, albeit of low quality, that pregnancy can result in accelerated clinical and functional progression or complications, such as pneumothorax and chylothorax, in patients with LAM.^158^ ^-^ ^164^ One retrospective study of pregnant patients with LAM produced results suggestive of disease progression during pregnancy, showing that the mean FEV_1_ fell from 77 ± 19% of predicted before pregnancy to 64 ± 25% of predicted after pregnancy, whereas the mean DL_CO_ fell from 66 ± 26% to 57 ± 26% of predicted, respectively.^159^ Spontaneous pneumothorax occurs in 25-30% of patients during pregnancy and can be the initial manifestation of the disease.^159^ ^,^ ^161^

During pregnancy, it can be necessary to take an invasive approach to LAM-related pleural complications, such as chest tube drainage, pleurodesis, and pleurectomy.^160^ Pregnancy can also provoke extrathoracic complications of LAM, such as growth, rupture, and bleeding of renal angiomyolipomas, as well as increased volume of abdominal or pelvic lymphangioleiomyomas, and chylous ascites.^160^ ^,^ ^162^ ^,^ ^163^ ^,^ ^165^ The risk of obstetric complications, such as premature birth, fetal growth restriction, and spontaneous abortion, is also elevated in LAM.^158^ ^,^ ^162^ ^-^ ^164^ There is as yet no consensus regarding the most appropriate mode of delivery for women with LAM.^162^ However, pregnancy can proceed without relevant complications for the fetus or the patient with LAM, especially if the patient is stable and has normal or only mildly altered lung function.^160^ ^,^ ^163^ The factors determining a higher risk of pregnancy-related complications in LAM have not yet been definitively established.

The safety of mTOR inhibitors during pregnancy in LAM has not yet been established, and they are classified as category C; that is, they have unknown fetal teratogenicity and their use is not an absolute contraindication.^166^ There have been reports describing the use of sirolimus during pregnancy, without fetal complications.^163^ ^,^ ^164^ ^,^ ^167^ ^,^ ^168^ It is suggested that mTOR inhibitors be discontinued at least 12 weeks in advance and that their use be avoided during pregnancy, especially in the first trimester, as well as during breastfeeding.^164^ ^,^ ^168^ However, their discontinuation during pregnancy can lead to increased dyspnea, as well as hypoxemia and pneumothorax, as well as the worsening of extrapulmonary complications. In this context, in patients with advanced or progressive pulmonary impairment, it is possible to consider starting or maintaining sirolimus, preferably in low doses (≤ 1 mg/day), preferably from the second trimester of pregnancy. Therefore, the indication for starting or maintaining sirolimus during pregnancy must be individualized, and additional studies are needed in order to establish its efficacy and safety in this context.

Counseling to pursue or avoid pregnancy should be individualized, based on the clinical and functional status of the patient, their history of pneumothorax, chylothorax, and renal angiomyolipoma, as well as their need for sirolimus, taking into consideration their desires, cultural background, spiritual beliefs, and life goals. Patients should be informed of the risk of gestational complications, for themselves and the fetus. Serial PFT is recommended during pregnancy, and its frequency should be individualized. For pregnant women with LAM-TSC, genetic counseling is also recommended.

### Contraception and hormone replacement therapy

Female hormones, especially estrogen, are involved in the pathophysiology and development of LAM.^19^ ^,^ ^169^ The fact that it occurs predominantly in women of reproductive age and the slowing of lung function decline after menopause, together with reports of disease progression after exogenous estrogen supplementation and during pregnancy,^19^ ^,^ ^170^ support this hormonal effect. Although treatment with several hormonal agents, including estrogen modulators, progesterone, aromatase inhibitors, and gonadotropin-releasing hormone analogues, have been evaluated for the management of LAM, as has oophorectomy, none of those therapies have yielded consistent results in terms of their effects on disease progression.^19^ ^,^ ^129^ ^-^ ^131^ ^,^ ^171^

Hormone replacement therapy, estrogen-containing contraceptives, and infertility treatment should be avoided in patients with LAM because of the potential risk of disease progression and of the development of pulmonary and extrapulmonary complications. Topical estrogen for the treatment of vaginal atrophy can be considered.^19^ ^,^ ^169^ ^,^ ^172^

The options for contraceptive methods in patients with LAM include copper or progesterone intrauterine devices, progesterone via subcutaneous or oral implant, partner vasectomy, and barrier devices, and the choice among those options should be individualized.^19^

### Osteoporosis

Reduced bone mineral density occurs in up to 70% of patients with LAM and is correlated with age and disease severity, probably related to reduced physical activity in patients with dyspnea and functional limitation, as well as to natural or induced menopause and the use of corticosteroids in transplant recipients.^173^

Although hormone replacement therapy is associated with an increase in bone mineral density, it is contraindicated in LAM because of the risk of progression related to estrogen use. Periodic bone densitometry is recommended in LAM, particularly in menopausal patients and those with greater functional impairment. Treatment with calcium, vitamin D, and bisphosphonates is indicated in patients with osteoporosis, in patients with osteopenia associated with severe functional impairment, and in patients on the transplant waiting list. Resistance and strength training should be encouraged.^173^

## PROGNOSIS

The natural history and prognosis of LAM remain incompletely understood; most analyses of the topic have been retrospective studies, and methodologies have been heterogeneous across studies.^174^ Although early studies reported that the median survival among patients with LAM was 8-10 years after diagnosis, data obtained more recently have suggested a better prognosis.^170^ ^,^ ^174^ ^-^ ^176^ Studies conducted in the United States have demonstrated a transplant-free survival rate of over 20 years in LAM,^30^ ^,^ ^174^ whereas a study conducted in the United Kingdom showed that the 10-year survival rate from symptom onset was 91%.^100^

Clinical, functional, laboratory, and CT variables have been evaluated as potential prognostic factors in LAM. Menopause reduces the rate of decline in FEV_1_ and the risk of progression to death or lung transplantation.^20^ ^,^ ^130^ One of the studies conducted in the United States supported this concept, showing that premenopausal women had a faster rate of decline in lung function and a higher risk of death, as well as being more likely to require lung transplantation, in comparison with those who were postmenopausal.^30^ In women with LAM, pregnancy and infertility treatment increase the risk of worsening lung function and the occurrence of complications, such as pneumothorax and growth of renal angiomyolipomas, as well as the risk of premature delivery and spontaneous abortion.^7^ ^,^ ^30^ ^,^ ^177^ ^,^ ^178^

Functional evolution associated with the presence of TSC has controversial results. Another study conducted in the United States demonstrated no difference in functional decline between patients with LAM-TSC and those with the sporadic form,^32^ whereas a recent study conducted in Brazil showed that LAM-TSC is associated with a smaller longitudinal reduction in lung function.^3^

The evaluation of lung function is useful for establishing baseline severity and facilitating the monitoring of disease progression. A FEV_1_ < 70% predicted, a reduced FEV_1_/FVC ratio, elevated TLC, and reduced DL_CO_ are predictors of a poor prognosis.^35^ ^,^ ^130^ The rate of decline in FEV_1_ is associated with the extent of pulmonary cysts on chest CT.^35^ Patients with a significant bronchodilator response tend to have more severe disease and more accelerated functional decline, possibly related to greater cell proliferation.^96^ ^,^ ^130^

Serum VEGF-D has potential prognostic relevance in LAM, with elevated levels being associated with the severity of the pulmonary impairment, reduced exercise tolerance, and the presence of lymphangioleiomyomas or lymphadenopathy.^30^ ^,^ ^85^ ^,^ ^179^ However, there is still a lack of evidence that elevated VEGF-D levels are associated with a higher risk of death or lung transplantation.^30^ The extent of pulmonary cysts on CT is associated with the severity and rate of decline of pulmonary function in LAM,^30^ ^,^ ^49^ thus constituting another method of prognostic assessment.

## FOLLOW-UP

If possible, patients with LAM should be monitored at referral centers. During follow-up, the severity, rate of progression, complications, and emergence of comorbidities can be identified, as can the tolerance and effectiveness of treatment. The frequency and interval of consultations and ancillary examinations should be individualized and are influenced by the clinical picture, severity, and rate of progression of the disease, as well as the need for monitoring of the treatment. It is generally recommended that consultations be conducted every 3-6 months in the first year after diagnosis and, if the condition is stable, every 6-12 months thereafter.

In patients with LAM, disease progression should be monitored with serial PFT. Simple spirometry with bronchodilator testing is recommended every 3-6 months in the first year after diagnosis; then every 3-12 months depending on progression. Annual plethysmography and DL_CO_ measurement are also recommended. Chest CT scans are recommended every 2-3 years if the condition is stable. If there is progression or emergence of new symptoms, functional decline, or suspected complications such as pneumothorax, CT scans should be performed immediately and their frequency should be reassessed.

In the monitoring of renal angiomyolipomas and abdominal lymphangioleiomyomas, it is recommended that abdominal MRI or CT be performed every year, every 2 years, or immediately if progression or complications are suspected. For screening, abdominal ultrasound can be performed every 2-3 years for patients without such manifestations.^27^

For patients taking sirolimus, it is recommended that laboratory tests be performed 2-3 weeks after starting the medication or changing the dose, then every 3-6 months, and those tests should include a complete blood count, creatinine, transaminases, alkaline phosphatase, gamma-glutamyltransferase, bilirubin, total cholesterol (and fractions), triglycerides, sodium, potassium, magnesium, calcium, phosphorus, blood glucose, and serum sirolimus.^27^ For elective surgeries, it is recommended that sirolimus be discontinued 1-2 weeks before and resumed 2 weeks after.

The need for breast cancer screening as indicated for each age group should also be emphasized. One retrospective study demonstrated a higher risk of estrogen receptor-positive breast cancer in LAM.^180^ In addition, a recent study conducted in Japan showed an increased risk of lung cancer in nonsmoking patients with LAM, suggesting that attention be paid to this aspect during follow-up.^181^

For patients with LAM who have extrapulmonary manifestations, specialized monitoring by multidisciplinary team (including a nephrologist, a neurologist, a dermatologist, and others) is also recommended. In cases of pneumothorax and chylothorax, the participation of a thoracic surgeon facilitates the therapeutic decision-making. For patients with impaired quality of life and mental health challenges, it is important to provide psychological evaluation and support services.

## PERSPECTIVES

### New biomarkers

There is a need to expand the spectrum of biomarkers, which are important for diagnostic, prognostic, and therapeutic response assessment, to those other than VEGF-D, several of which have recently been studied in LAM, although none are yet used in the clinical routine.

In patients with LAM, MMPs, especially MMP-2, are involved in the degradation of the extracellular matrix and cystic destruction of the lung parenchyma.^182^ Another promising marker is fibroblast growth factor 23 (FGF23), a protein secreted by osteocytes that is essential for maintaining serum phosphate homeostasis, which is dysregulated in human diseases that affect bone mineral density. Chronic lung diseases such as COPD and idiopathic pulmonary fibrosis have been associated with FGF23. Serum FGF23 levels differentiate LAM patients from controls, the levels being higher in the former, and lower FGF23 levels are associated with reduced DL_CO_.^183^

Studies employing machine learning are promising. An analysis using this methodology on serum samples from study participants found that the biomarker combination of VEGF-D + EFNA4 + IGHD + GDNF + TKT showed high accuracy in predicting a decline in FEV_1_ within 6 months.^184^

### Use of mTOR inhibitors in patients with normal lung function

The use of sirolimus only in patients with LAM with functional impairment, as established in one study, somewhat limits the real-world prospects for treatment.^38^ The medication appears to have similar stabilization potential in patients with different degrees of severity, even among postmenopausal patients, suggesting its benefit in mild disease.^20^ In addition, maintaining even low serum levels of sirolimus appears to be sufficient for a favorable effect on PFT and its stabilizing action, even before the lung damage has been reversed, making it attractive to initiate the treatment earlier, without waiting for significant functional impairment.^89^

It has been suggested that sirolimus is best used when there is loss of lung function (typically ≥ 90 mL/year) and that stable patients, especially menopausal patients, can be followed without treatment, even if they have PFT results indicate of impairment.^28^ The use of low-dose sirolimus in patients with normal PFT results has the potential to prevent long-term complications and is the subject of an ongoing clinical trial.^185^ At this point, additional factors such as age, menopausal status, extrapulmonary manifestations, recurrent pneumothorax, and significant cell proliferation on biopsy can be taken into account in defining the best treatment strategy.

### New treatments

It is essential that new pharmacological therapies in LAM be investigated, given that treatment with sirolimus is not definitive, can provoke adverse events, and needs to be maintained continuously, as well as the potential for the development of drug resistance. However, conducting clinical trials in LAM poses many difficulties, including a lack of investment to recruit patients for international multicenter studies, clinical heterogeneity, the rarity of the disease, and ethical questions regarding the randomization of patients to a control group, given that a number of drugs have been approved for the treatment of LAM.^186^ New medications have been studied as potential therapeutic options in LAM, although their use is not yet recommended in daily practice.

Drugs that inhibit autophagy, such as hydroxychloroquine and chloroquine, have proven effective for tumor reduction and inhibition of cell survival when combined with sirolimus. A phase I study demonstrated safety, good tolerance, and favorable effects of this combination.^187^ The combination of resveratrol, which acts on the autophagy process, with sirolimus demonstrated good tolerance and safety, with reduced VEGF-D levels and improved quality of life.^188^

Nintedanib, an intracellular inhibitor of tyrosine kinases such as the platelet-derived growth factor receptor, which is active in LAM lesions, was investigated in a phase II trial as a possible second-line therapy for LAM patients who are refractory to or present with adverse events due to sirolimus use, demonstrating good tolerance but without improvement in FEV_1_.^189^

Although other medications, such as nitazoxanide, aromatase inhibitors, immunotherapeutics, and simvastatin have been studied, there are still no consistent results supporting their use in LAM.^190^ ^-^ ^193^

## FINAL CONSIDERATIONS

Defined as a low-grade neoplasm, LAM is considered to have metastatic potential, although the origin of its cells is as yet unknown. Several advances have been made in the last two decades, mainly in relation to pathophysiology and management, such as the use of serum VEGF-D levels in the investigation, a systematic diagnostic approach, and the use of mTOR inhibitors as a therapeutic option. The aim of this document was to present the main points related to the approach to LAM, including some practical aspects for its management and for improving the daily lives of patients with the disease. Given the multisystemic nature of the disease, there is a need for a multidisciplinary approach. It should be borne in mind that there is still no definitive, curative treatment for LAM, and that there is a need for new, noninvasive tools for its diagnostic confirmation, the development of which is expected in the near future.
